# Use of permeability surface area-product to differentiate intracranial tumours from abscess

**DOI:** 10.2349/biij.5.1.e6

**Published:** 2009-01-01

**Authors:** N Ramli, K Rahmat, E Mah, V Waran, LK Tan, HT Chong

**Affiliations:** 1 Department of Biomedical Imaging, Faculty of Medicine, University of Malaya, Kuala Lumpur, Malaysia; 2 Department of Surgery, Faculty of Medicine, University of Malaya, Kuala Lumpur, Malaysia; 3 Department of Medicine, Faculty of Medicine, University of Malaya, Kuala Lumpur, Malaysia

**Keywords:** CT perfusion, permeability surface area-product, brain abscess, necrotic tumours

## Abstract

**Background and Purpose:**

Clinical and radiological findings of intracranial abscesses may mimic the findings of brain tumours and vice versa. However, the discrimination is of great clinical importance in planning treatment and in following prognosis and response to therapy. This study evaluates the Computed Tomography (CT) perfusion parameters, especially the permeability index, with the aim of evaluating the usefulness of dynamic CT perfusion imaging as an alternative tool to differentiate necrotic brain tumours and intracerebral abscesses.

**Materials and Methods:**

A total of 21 patients underwent perfusion CT study and were divided into 2 groups: Group 1, patients with necrotic brain tumours (n=13); and Group 2, patients with cerebral abscesses (n=8). The mean perfusion parameters were obtained from the enhancing part of the lesion. The relative ratios were then calculated by using the results from mirrored regions within the contralateral hemisphere as reference.

**Results:**

The results of this study showed that there was significant difference in the relative permeability surface values between necrotic brain tumours and cerebral abscesses (p=0.005). By applying the ROC curve, a value of 25.1 for rPS was found to be the best estimate to distinguish necrotic brain tumours from cerebral abscesses with a specificity of 88 % and sensitivity of 70 %.

**Conclusion:**

CT perfusion, especially permeability surface, may allow for better differentiation of cerebral abscesses from brain tumours, making it a strong additional imaging modality in the early diagnosis of these two entities.

## INTRODUCTION

It has been written that tumour angiogenesis differs from the infective process in both histological, biochemical markers and perfusion study. Tumour angiogenesis is characterised morphologically by an increase in the number of blood vessels, including new capillaries, capillary sprouts, non-endothelialised capillaries, and arterio-venous shunts. Some of these abnormal vessels tend to be leaky, which allows large molecules such as plasma protein to enter the stroma to form the matrix for new vessels.

Intracranial infection and abscess formation is a progressive and dynamic process with its own vascular changes. Until recently, options for cerebral perfusion measurements were restricted to positron emission tomography (PET), single photon emission computed tomography (SPECT) or xenon CT. New sequences and advances in Magnetic Resonance Imaging (MRI) such as MR perfusion, in addition to MR spectroscopy, diffusion-weighted echo planar sequences (DWI) and apparent diffusion coefficient map (ADC map) have helped to differentiate intracranial abscesses and brain tumours. However, the application of MRI is sometimes limited by its availability, expense, post surgical artifacts and the 15-20% patients who are unable to undergo or tolerate an MR in acute setting. The introduction of dynamic CT perfusion capable of measuring brain haemodynamics with short acquisition time, using multislice CT available in most radiology departments, has been well-received. We conducted a CT perfusion study with emphasis on permeability surface area-product to ascertain its possible role in differentiation of cerebral infection and brain tumours. We believe that the findings of this study will have valuable clinical implications on patient management.

Permeability surface area-product (PS) measures the permeability of the blood brain barrier to contrast material. Permeability is related to the diffusion coefficient of the contrast agent in the assumed water-filled capillary endothelieum. It is expressed in the equation: PS = -CBF.ln(1-E), where E is the extraction fraction, that is the fraction of contrast material that leaks into the extravascular space from the intravascular space [1].The unit for PS is ml/100gm/min.

## METHODOLOGY

This was a cross-sectional study of 25 patients with a single intracranial necrotic lesion in the period of November 2004 till January 2007. Each patient underwent an unenhanced CT scan and a CT perfusion of the brain. Ethical clearance was approved by the institutional review board and informed consent was obtained by each study participant.

Our consecutive series were selected from patients with known intracranial necrotic lesions of either cerebral tumour or abscess who were referred to our radiology department for CT scan of the brain. There were a total of 25 patients who underwent the standardised perfusion scan in this study; however 4 were unsuccessful, with 2 due to severe motion artifacts and the other 2 due to technical errors. The successful subjects were divided into 2 groups: Group 1, patients with brain tumours (n=13), 2 who had glioblastoma multiforme, 3 who had primary lymphoma (the diagnosis of this group of patients were obtained by biopsy) and another 8 who had a known primary carcinoma and secondaries to the brain (2 from Ca lung, 2 from Ca breast, 1 from Ca stomach, 1 from Ca rectum, 1 from Ca cervix and 1 from endometroid Ca). Group 2 were the patients with infection, where the diagnosis was achieved either by culture of the sample obtained during surgical drainage or culture of the cerebrospinal fluid obtained by lumbar puncture, (n=8, 2 toxoplasmosis, 1 staphylococcal infection and 1 streptococcal infection from septicaemia, 2 were of mixed culture and 2 were undetermined organism given empirical antibiotic treatment).

An unenhanced CT scan of the whole brain was first performed using a 16-slice CT scanner (GE LightSpeed Plus, Milwaukee) on all patients whereby 2.5mm thick transverse sections through the posterior fossa and 5mm thick transverse sections through the supratentorial region were obtained. The images were then reviewed. The intracranial lesion was then identified and a 10mm slice which has the most representation of the lesion was then selected. Acquisition of CT perfusion data was then performed on the region of interest following a bolus injection of 40cc of ultravist (iopramide: 370mgI/ml strength contrast media) at 4cc per second. All perfusion scans were done using the LightSpeed protocol where repeated scanning of four adjacent 5mm transverse sections was performed with a 45 second cine with 1 second interval, acquired at 80kvp using 200mAs. A 25cm field of view was used for all scanning. Data were then retro reconstructed into 10mm at 0.5sec interval with a matrix of 512 x 512 pixels.

**Figure 1:**
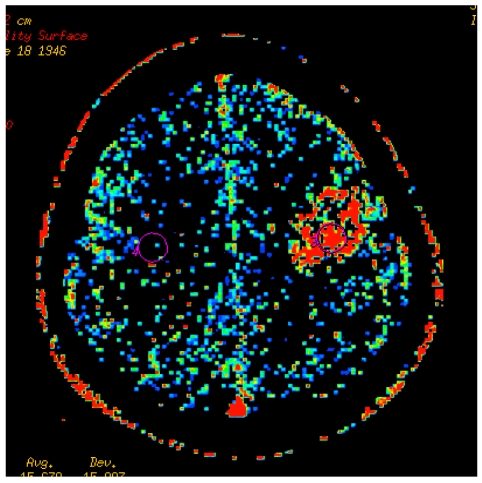
PS map of the patient with necrotic lesion.

All the scans were then transferred to the imaging workstation (AW4.0; Advantage Windows; GE Medical Systems, Milwaukee, Wisconsin) and analysed using the CT perfusion software by GE Medical Systems. The colour-coded maps of cerebral perfusion parameters were computed when the contrast enhancement curves are generated. ROIs in the CT brain images were then drawn in the lesion with the greatest enhancement, while avoiding the nearby vessels. In the corresponding contralateral normal regions, another ROI was also generated as a control region (Figure 1). The analysis software allowed ROI drawn on the reference image to appear simultaneously on all the perfusion maps. This procedure is repeated twice for each lesion and the mean values were calculated. The size of the ROIs was standardised in all the perfusion samples to be within 115-125mm².

Relative perfusion of PS was calculated by using the results from mirrored regions within the contralateral normal hemisphere as reference. (The reason why relative value is used instead of absolute values is in the Discussion section).

$$\text{Relative PS = }\frac{\text{Absolute PS value of the lesion}}{\text{Absolute PS of the corresponding contralateral side}}$$

The parameters of permeability surface (PS: ml/100g/min) were evaluated. Statistical analysis was performed using SPSS 13.0 Scientific Software Packages (Chicago, IL). Standard descriptive statistics, such as maximum, minimum, mean ± SD, were calculated. Mann-Whitney U test, a nonparametric independent test, was used to determine the statistical differences of the data obtained. Statistical significance was declared at the p< 0.05 level.

## RESULTS

The Mann-Whitney test revealed that there was no significant statistical difference in rCBF (p=0.169), rCBV (p=0.192) and rMTT (p=0.385) between necrotic brain tumours and cerebral abscess.

**Figure 2:**
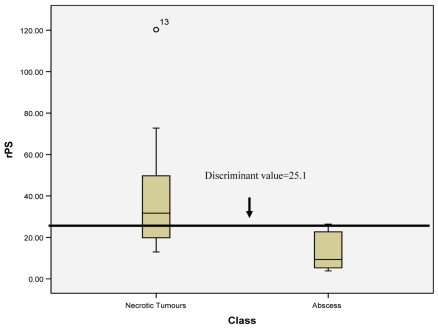
A box plot graph showing the rPS values in cerebral tumours and cerebral abscesses and the discriminant rPS value between these two groups.

However, there was significant statistical difference in rPS value between necrotic brain tumours and cerebral abscesses (p=0.005). By applying the ROC curve, a value of 25.1 for rPS was found to be the best estimate to discriminate cerebral tumours from cerebral abscesses with a specificity of 88%, sensitivity of 70% ( Figure 2). This means that intracranial necrotic lesion with rPS value of more than 25.1, had an 88% chance of being a tumour rather than an abscess.

## DISCUSSION

Intracranial abscesses are life-threatening medical emergencies with a mortality rate ranging from 30-70% [2, 3]. Delay in proper medical therapy can be devastating. At the same time, differentiating intracranial abscess from many other disease processes can be difficult because of non-specific clinical and radiographic findings. The classical ring-enhancing appearance of an intracranial abscess can be mimicked by several other entities, most notably by a necrotic tumour. A report by Erdagon *et al* [4] concluded that diffusion MRI may be sufficient in differentiating abscesses from cystic tumours, and that CBV map images may aid in differentiation by demonstrating lesion vascularity. It was found that the vascular portions of abscesses have a decreased rCBV while the peripheral portions of malignant cystic tumours had higher vascularisation and higher rCBV.

There were studies describing perfusion parameters, mainly CBF and CBV, in the pathologic evolution of brain abscesses. Soon after inoculation with an offending organism, increases in CBF and CBV were observed. These changes were seen 24 hours after innoculation, and increased to a maximum between 48-72 hours later, at which point perfusion changes appeared to plateau within the region of infection. Increased blood brain barrier permeability in the infected region was rapidly manifested as both regional oedema and minimal ill-defined areas of contrast-enhancement. The peak increase in BBB permeability correlated with the appearance of an enhancing ring surrounding the region of cerebritis. The most peripheral part of the ring was enhanced first, with the more central portion enhancing later, occasionally completely filling in the abscess. Thus, during the phase of cerebritis, perfusion changes were relatively non-specific, and could be mimicked by viral encephalitis, lymphoma or primary tumours [5].

In the early stage of abscess formation, the collagen capsule is incomplete, but the perilesional oedema would become less extensive. As the vascular proliferation becomes maximal, interestingly, it does not extend beyond 1 mm of the inflammatory region in the necrotic centre. This may not be apparent on conventional CT as the ring of contrast enhancement appears thick. With dynamic-acquisition, increased rCBV may be present in a very thin rim surrounding the necrotic centre; however, within the wider area of the abscess wall, decreased rCBV is observed [6, 7]. This in contrast with the pattern seen in ring-enhancing lesions such as glioblastoma and lymphoma, where more angiogenesis at the tumour margin is observed and manifested by increased rCBV.

A study depicted by Ernst *et al* had used perfusion images in distinguishing acquired immunodeficiency syndrome (AIDS) lymphoma from toxoplasmosis [8]. This distinction frequently cannot be made routinely by conventional MR or CT imaging alone. CBV was found to be decreased in each of the toxoplasmosis lesions, whereas all the active lymphomas displayed regions of increased CBV.

Malignant lesion has been shown to have a high permeability to macromolecules due to the presence of large gaps between or fenestration of endothelial cell, incomplete basement membrane and high vascular tortuosity [9]. The high vascular permeability associated with high grade gliomas support the fact that these neoplasms interrupt the bloodbrain barrier ([10-13]. There have been multiple studies looking at MR perfusion values in tumour grading; in particular demonstrating that higher CBV value is associated with higher grade [14, 15] tumours. However MR usage has its drawbacks, especially in very ill or debilitated patients.

With recent advances in multi-slice CT technology and friendly commercial software, it is now possible to perform perfusion studies on intracranial lesions. Recent studies looking at the use of CT perfusion index such as CBV and PS found that these parameters provide useful information for grading gliomas and might have the potential to significantly impact clinical management and follow-up of cerebral gliomas. In these studies, permeability surface area product has been shown to be particularly helpful ([16-18]. There have been studies that demonstrated increased conspicuity of tumours when using PS index compared to CBV and CBF [19,20].

In this study, the permeability maps were also more visually conspicuous than the CBF and CBV maps in showing up the intracranial tumours. Comparing the PS in tumours to that in normal tissue, this study showed that tumours yielded an average value that was 30 times greater. This study concentrated on differentiation of single space occupying lesions, and did not look into the grading of gliomas. However, it was found that the CBV value was not helpful in the differentiation of primary tumour from metastases.

One of the limitations of CT perfusion is high radiation exposure. Even when popular low-dose protocols are used, (80 kVp, 200mA, 1s per rotation and 50 rotations) the volume CT dose index (CTDIvol) is estimated to be 500-700mGy with effective dose of 2.8 to 7.8 mSv. This value is approximately 10 times higher than the diagnostic reference level of the brain CT and is roughly equal to that of cerebral angiography. Currently, 80kVp is widely accepted as an appropriate tube voltage because the radiation exposure can be reduced to one-third of that with 120kVp and the contrast between iodine contrast agents and brain tissues can be enhanced [21,22]. Tube current of 200mA is also optimal as excessive reduction would induce increase in quantum noise from X-ray photons, resulting in serious errors in analysis. Scanning with slow rotation speed up to 1.5s can reduce tube current to two-thirds while maintaining the signal-to-noise ratio. The intermittent scanning at every second can also reduce the dose by 50%. In a report by M. Sasaki et al., a CTDI vol of approximately 100-170mGy can be achieved by utilising an ultra low-dose protocol (80kVp, 30-50mA, 1.5s/rotation and 30 rotations) and a sophisticated filter, which selectively removes quantum noises while preserving image details [23].

The other limitations and controversies are the current use of low-molecular weight contrast for human subjects. It is hypothesised that contrast agents with low molecular weights may leak relatively easily from blood into the interstitial and therefore lead to overestimation of permeability [24]. Alternatively, contrast agents with high molecular weights leak blood more slowly, and transfer into interstitial matrix is limited in relation to tumour blood flow. It is postulated that the use of low molecular weight contrast in this study (Iopromide=791.12) may affect the permeability index on the normal contra-lateral side, which should be negligible. In the absence of a nanoparticulate contrast agents which is synthesised to a precise size to allow a more accurate and independent monitoring of tumor vessel permeability and vascular volume as suggested by Choyke [25], it was proposed to normalise the PS by using relative PS measurements. In this study, this method has been proven to provide statistical significant results to differentiate abscess from necrotic tumours.

The other disadvantage is the narrow scanning range and therefore narrow anatomic coverage, which is restricted to 20-40mm even in novel multi-detector row CT . Combined with faster tube rotation speeds, table-toggling techniques, or increased detector elements perfusion, CT studies covering the whole brain may become possible [26,27].

Further studies are needed to better establish the precise sensitivity and specificity of CT perfusion imaging in relation to the time course of the perfusion parameters changes following the onset of cerebral abscess. The low number of cases in this study may be a limitation. Further studies with larger numbers, including more variety of tumours and abscesses at various stages, should be conducted.

**Table 1:** Patients with intracranial necrotic brain neoplasm — Absolute and relative CBF, CBV, MTT and PS values

**Pt**	**Diagnosis**	**CBF**		**CBV**		**MTT**		**PS**	
		Absolute	Relative	Absolute	Relative	Absolute	Relative	Absolute	Relative
1	GBM	29.63	0.33	1.98	0.57	2.59	0.48	31.65	25.52
2	GBM	22.75	0.50	2.38	1.95	3.42	0.47	12.21	18.78
3	Ca Lung	148.59	2.66	9.40	2.65	5.04	1.04	24.76	37.47
4	Ca Lung	559.70	3.68	29.26	3.40	3.64	.99	64.82	31.68
5	Ca Breast	35.33	1.82	2.99	2.84	7.57	1.42	15.64	72.76
6	Ca Breast	78.14	1.26	5.74	1.89	5.22	1.55	21.66	12.95
7	Ca Stomach	129.54	2.25	5.50	1.03	7.20	2.07	30.99	31.69
8	Colorectal Ca	96.12	1.44	5.37	1.25	6.18	2.07	36.33	49.75
9	Ca Cervix	40.09	1.38	2.19	1.47	4.89	1.59	6.11	14.21
10	Endometroid Ca	103.83	1.73	5.62	1.34	6.21	1.99	26.33	19.80
11	lymphoma	20.60	0.71	1.61	0.62	4.69	0.88	3.08	61.60
12	lymphoma	27.91	0.77	1.96	1.15	3.24	.58	7.35	45.94
13	lymphoma	35.33	1.82	2.99	1.50	7.57	1.42	15.64	120.31

**Table 2:** Patients with cerebral infection — absolute and relative values of CBF, CBV, MTT and PS values.

**Pt**	**CBF**		**CBV**		**MTT**		**PS**	
	Absolute	Relative	Absolute	Relative	Absolute	Relative	Absolute	Relative
1	45.06	1.22	2.49	1.58	3.22	1.01	3.32	5.63
2	56.48	1.92	3.36	2.18	4.64	.82	5.38	20.69
3	13.46	0.40	0.73	0.39	7.07	1.62	1.05	5.00
4	112.90	1.84	6.13	1.69	3.95	0.95	4.99	7.45
5	70.77	1.96	4.96	2.23	5.31	1.08	5.19	24.70
6	153.23	4.18	6.42	3.22	2.89	.69	7.01	3.81
7	82.61	2.45	7.05	3.36	5.53	1.17	7.39	26.40
8	135.00	2.95	6.81	2.46	3.41	.79	4.14	11.19

**Table 3:** Comparison of perfusion values using between necrotic tumours and abscess.

	**rCBF**		**rCBV**		**rMTT**		**rPS**	
	Tumour	Abscess	Tumour	Abscess	Tumour	Abscess	Tumour	Abscess
**N**	13	8	13	8	13	8	13	8
**Mean**	1.57	2.12	1.66	2.14	1.27	1.02	41.7	13.1
**Median**	1.44	1.94	1.47	2.21	1.42	0.980	31.6	9.32
**Std. Deviation**	0.934	1.13	0.856	0.952	0.581	0.291	29.8	9.35
**Minimum**	0.33	0.40	0.57	0.39	0.47	0.69	13.0	3.81
**Maximum**	3.68	4.18	3.40	3.36	2.07	1.62	120	26.4
**p-value**	0.169		0.192		0.385		0.005	

## CONCLUSION

To the best of the authors' knowledge, this is the first study in which the correlation between PS in differentiation of intracranial tumours and abscess was investigated. Overall there was a much higher value in PS for tumours compared to abscess. In clinical practice, when MR cannot be done for various reasons, CTP is a fast, easy and widely available alternative for differentiation of tumours and abscess. In particular, the rPS value above 25.1 would favour the diagnosis of cerebral tumours rather than intracranial infection.
